# Camptocormie et maladie de parkinson: une association ou une conséquence?

**DOI:** 10.11604/pamj.2015.20.339.6586

**Published:** 2015-04-09

**Authors:** Samia Frioui, Sonia Jemni

**Affiliations:** 1 Service de Médecine Physique et de Réadaptation Fonctionnelle, CHU Sahloul, Sousse, Tunisie

**Keywords:** Camptocormie, Maladie de Parkinson, Camptocormia, Parkinson disease

## Image en medicine

**Figure 1:**
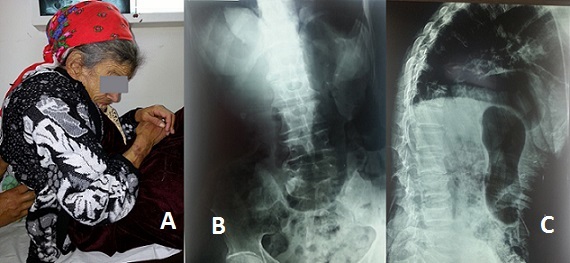
(A) anté flexion de la tête et du tronc; (B) radiographie du rachis dorsolombaire de face: inclinaison droite du rachis sans rotation des corps vertébraux; (C) radiographie du rachis dorsolombaire de profil: effacement de la lordose lombaire, exagération de la cyphose dorsale

La prévalence des troubles statiques rachidiens dans les syndromes extrapyramidaux est de 13 à 90 % selon les études. Les observations de camptocormie associée à la maladie de Parkinson restent rares. Il s'agit d'un trouble postural caractérisé par une flexion du tronc qui se manifeste à la station debout et est réductible en position couchée. C'est la complication orthopédique fonctionnellement la plus grave de la maladie de Parkinson. Elle altère gravement l'autonomie, s'accompagne de désordres posturaux et correspond très probablement à une dystonie du tronc. Mme M., âgée de 71 ans, connue porteuse d'une maladie de Parkinson depuis 14 ans sous traitement avec une mauvaise observance thérapeutique, était adressée en consultation de Médecine Physique et de Réadaptation Fonctionnelle pour escarres et perte de l'autonomie. La patiente présentait des troubles posturaux du tronc, un ralentissement psychomoteur et un alitement depuis huit mois. À l'examen clinique, il était observé une inclinaison rachidienne droite avec antéflexion, une hypertonie des muscles paravertébraux droits, une bradykinésie, un visage peu expressif et une voix assourdie. La radiographie du rachis retrouvait de face une inclinaison droite sans rotation des corps vertébraux et de profil un effacement de la lordose lombaire associé à une hypercyphose dorsale. Il n'y avait pas de tassement vertébral. Le bilan biologique était normal. La constatation de ce trouble postural sans argument radiologique ou biologique pour une étiologie précise, faisait évoquer une origine extrapyramidale. L'hospitalisation a permis une amélioration des escarres mais malheureusement la patiente n'a pas repris la marche. Figure 1

